# Analyzing effects on anterior open bite in twins by PLS-SEM and sobel test

**DOI:** 10.1007/s00784-024-05874-1

**Published:** 2024-08-15

**Authors:** Sinem Birant, Mert Veznikli, Yelda Kasimoglu, Mine Koruyucu, Atıf Ahmet Evren, Figen Seymen

**Affiliations:** 1 Faculty of Dentistry, Department of Pediatric Dentistry, Istanbul University- Cerrahpaşa, Istanbul, Turkey; 2 Faculty of Science and Arts, Department of Biostatistics, Koç University, Istanbul, Turkey; 3 Faculty of Dentistry, Department of Pedodontics, Istanbul University, Istanbul, Turkey; 4 Faculty of Science and Arts, Department of Statistics, Yildiz Technical University, Istanbul, Turkey; 5 Faculty of Dentistry, Department of Pediatric Dentistry, Altinbas University, Istanbul, Turkey

**Keywords:** Pediatric dentistry, Swallowing, Structural equation modeling, Clinical studies/Trials, Orthodontic(s)

## Abstract

**Objective:**

This study aimed to assess the different pathways between predictor factors such as zygosity, atypical swallowing, mouth breathing, breastfeeding and bottle feeding related to anterior open bite (AOB) in twins.

**Methods:**

The study was conducted in monozygotic (MZ) and dizygotic (DZ) twin children aged 3–15 years. AOB, atypical swallowing, mouth breathing, feeding type, duration of bottle use, and mouth opening status during sleep were recorded during oral examination. Partial least squares structural equation model (PLS-SEM) and sobel tests were performed to assess the total and indirect effects among the variables on AOB.

**Results:**

A total of 404 children (29.2% MZ;70.8% DZ) participated in this study. The effect of zygosity on mouth breathing in the PLS-SEM model was statistically significant. Conversely, it was determined that mouth breathing effected that atypical swallowing (*p* = 0.001). Atypical swallowing triggered AOB (*p* = 0.001). The atypical swallowing has a mediation effect between AOB and mouth breathing (*p* = 0.020). Mouth breathing causes atypical swallowing and therefore indirectly increases the likelihood of AOB. While breastfeeding decreases AOB incidence (*p* = 0.023), bottle feeding increases AOB incidence (*p* = 0.046). The sobel tests show that the fully mediator variable feature of mouth breathing is statistically significant in the negative relation between zygosity and atypical swallowing.

**Conclusion:**

The PLS-SEM model showed that mouth breathing triggers atypical swallowing and atypical swallowing triggers AOB. As a result of this chain of relationships, an indirect effect of zygosity on AOB was observed. According to sobel tests, zygosity has an indirect effect on atypical swallowing through mouth breathing, while mouth breathing has a positive indirect effect on AOB through atypical swallowing.

**Clinical relevance:**

This study identified the relationships between different factors and the presence of AOB. The findings of this study demonstrate in detail the relationships between AOB and zygosity, atypical swallowing, mouth breathing, breastfeeding and bottle feeding. Brestfeeding has a reducing effect on the frequency of AOB. Among the nutritional forms, breastfeeding ensures the proper development of the stomatognathic system by working the oro-facial muscles.

## Introduction

Malocclusion is associated with altered development of dental arches and are recognized as a public health problem [1]. It negatively affects the quality of life of the patient with its high prevalence, aesthetic and functional effects [2, 3]. Anterior open bite (AOB) is a type of malocclusion characterized by insufficient incisal contact due to the change in the development of the dental arches in the vertical plane [4, 5]. The etiological factors of AOB are classified as genetic and environmental [6]. Additionally, amelogenesis imperfecta, idiopathic condylar resorption, and ankylosis of the anterior teeth may be linked to an AOB [7]. AOB has a genetic component that is related to genes that generate craniofacial abnormalities [8]. Studies have discovered certain genes and signaling pathways involved in jaw growth and tooth eruption, and differences in these genes may contribute to the development of an open bite [9]. While genetic factors are related to facial skeletal patterns, environmental factors are related to dento-alveolar eruption disturbances, mouth breathing, non-nutritive sucking habits, diet, tooth loss, and early weaning [10, 11].

Muscle activity during breastfeeding provides physiological growth of the jawbone, and at the same time, the correct position of the tongue facilitates the development of proper swallowing technique [1, 4]. Bottle feeding changes muscle activity, with some muscles being hypoactive (orbicularis oris, masseter), overactive (jaw and cheek muscles), or incorrectly positioned (tongue). For this reason, bottle feeding is among the main risk factors for AOB formation [11–14].

Atypical swallowing, one of the common harmful habits in children, is another environmental risk factor affecting AOB [2]. Differential positioning of the tongue during swallowing affects dental position, dental arch and development of maxillary teeth [3]. In normal swallowing, the tip of the tongue rests on the palatal papilla, the dorsum of the tongue presses the palate, the teeth are in light contact, and the lips are closed to create negative pressure and perform a peristaltic movement to push the food bolus towards the esophagus. Atypical swallowing is accompanied by tongue or lip thrusting between anterior teeth [10]. However, the relationship between AOB and atypical swallowing has not been clearly revealed. Some studies indicate that atypical swallowing is associated with AOB, while other researchers suggest that atypical swallowing is the result of functional adaptation of an existing malocclusion [6, 15, 16].

This study was conducted with the aim of developing model to explain the predictor factors such as zygosity, atypical swallowing, mouth breathing, breastfeeding for first 6 months and bottle feeding related to AOB in 3–15 years old twins using partial least squares structural equation modeling (PLS-SEM).

## Materials and methods

The study was approved by Istanbul University Faculty of Medicine Clinical Research Ethics Committee (No: 214/278) and each patient’s parents provided written informed consent. This study is a human observational study and complies with STROBE guidelines.

Inclusion criteria: Monozygotic (MZ) and dizygotic (DZ) twin children between the ages of 3–15 years with complete incisor eruption.

Exclusion criteria: Children with a genetic or systemic disease, a mental problems, a history of orthodontic treatment, and ankylosis of anterior teeth, and with finger-sucking habits and lower lip sucking habits. Patients with unerupted or partially erupted incisors were excluded from the study.

Zygosity determination was confirmed for selected 100 twins whose gender was the same within each twin pair.

### Sample size calculation

A commonly used minimum sample size estimation method in PLS-SEM is the ‘10-times rule’ method. This method is based on the assumption that the sample size should be more than 10 times the maximum internal or external model connections pointing to any latent variable [17]. Because the total number of connections in the structural equation model we established is 17, a minimum sample size of 170 will be sufficient. The research was completed with a sample size of 404.

### Data collection

The clinical examination was performed by a trained pediatric dentist with 15 years of experience. The parameters to be examined for the clinical examination were standardized by preparing a form. The patients were called back after 2 weeks and the accuracy of the clinical findings was tested a second time by the same researcher. Intraobserver correlation was high.

Presence of AOB, atypical swallowing, and mouth breathing were recorded during oral examination. Data were collected with a questionnaire that was filled out by the parents, which included questions on the child’s clinical history, the type of feeding, the bottle use time, the state of the child’s mouth open during sleep. Breastfeeding was collected in months and categorized as yes (the child breastfed up to 6 months or more) and no (not breastfed or less than 6 months). Bottle feeding times were stated in months.

Mouth breathing status was evaluated during the clinical examination along with the patient’s adenoid facial characteristics such as labial incompetence, an open-mouthed posture, a high-arched palate, a narrow jaw and a long face, as well as questionnaires asked to the family [18].

Diagnosis of atypical swallowing was made by direct observation of the lingual interposition between the anterior teeth. AOB was defined as a lack of vertical overlap of the incisors in the occlusal position, and it was discovered when the anterior teeth did not touch a space between the incisal edges of the maxillary and mandibular teeth.

### Statistical analysis

**Fig. 1:**
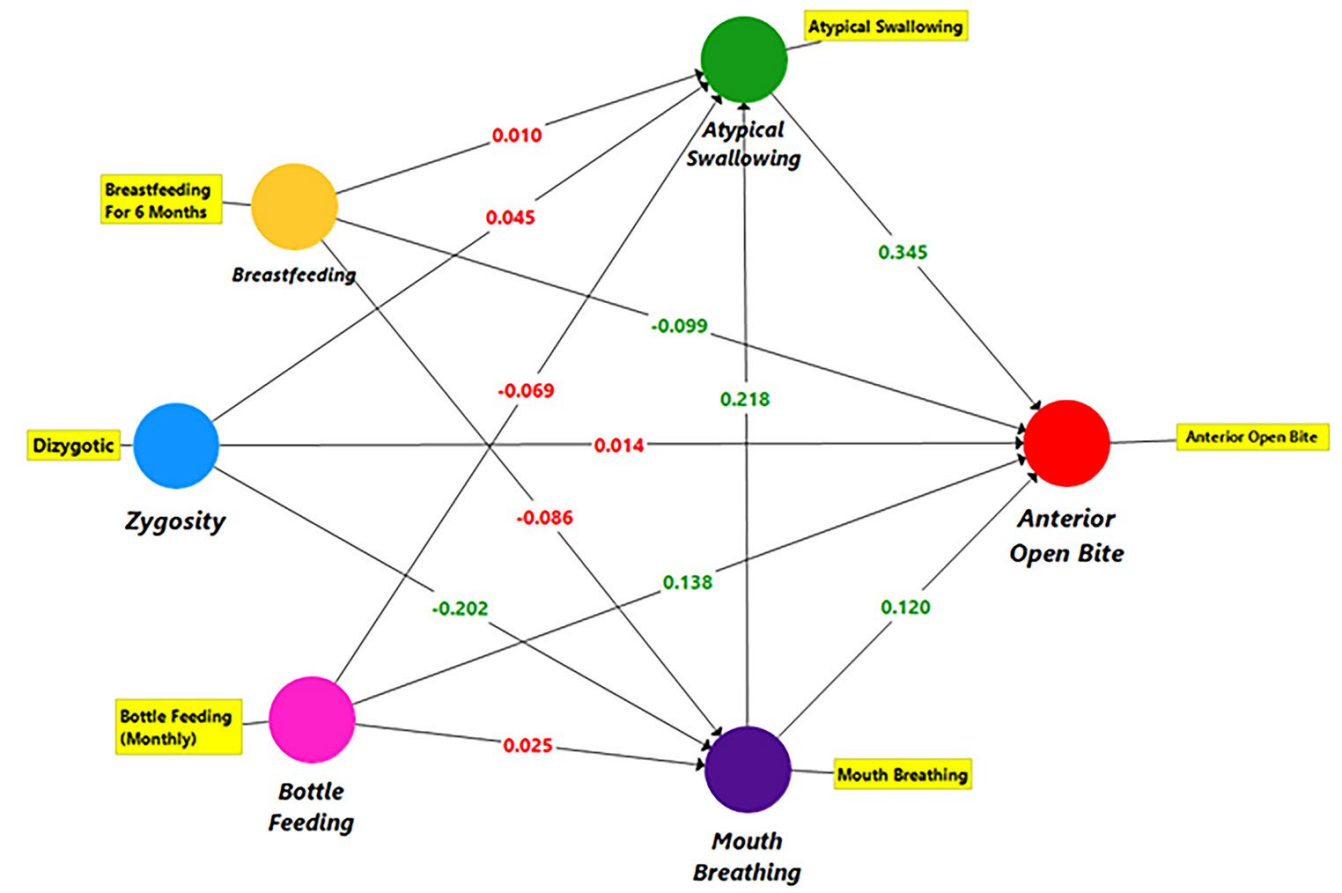
SmartPLS path model estimation shows path coefficients

Data from 404 twins were analyzed using the SmartPLS 3-Structural Equation Modeling (SEM) technique. In addition, datawere analyzed using IBM SPSS statistical program version 26. Figure 1 illustrates the conceptual model and relationships of the constructs used in this study based on the AOB model.

In established models, the effects between zygosity, breastfeeding, bottle feeding, mouth breathing, atypical swallowing and AOB were investigated. The tested hypotheses are:

- Zygosity has a significant effect on:
  - Mouth breathing.
  - Atypical swallowing.
  - AOB.
  - Indirectly on AOB.
  - Indirectly on atypical swallowing.

- Bottle feeding has a positive and significant effect on:
  - Mouth breathing.
  - Atypical swallowing.
  - AOB.

- Breastfeeding has a negative and significant effect on:
  - Mouth breathing.
  - Atypical swallowing.
  - AOB.

- Mouth breathing has a positive and significant effect on:
  - Atypical swallowing.
  - AOB.
  - Indirectly on AOB.

- Atypical swallowing has a positive and significant effect on AOB.

To test these hypotheses, a PLS-SEM was designed firstly (Fig. 1). The model was run with the bootstrap routine using the consistent PLS algorithm (PLSc). In PLS-SEM, non-parametric bootstrap, a second generation multivariate statistical method, is used to calculate estimated standard error values and t statistics and confidence intervals. It is recommended to resample 5000 times [19]. The model was run with 10,000 resamples and 5000 maximum iterations.

The same research model was then discussed with logistic regression models. In the logistic regression analysis, the input method was used and at the same time, the goodness of fit of the models was checked with the Hosmer-Lemeshow test and it was observed that the goodness of fit was achieved in all of the models (*p* > 0.05).

In the last part, Sobel, Aroian and Goodman mediation analysis were performed using the coefficient and standard error values obtained from the logistic regression models. Test values were performed using the Quantpsy online interactive tool at the web address http://quantpsy.org/sobel/sobel.htm [20]. The significance level was given as 0.05 in all analyzes.

## Results

A total of 404 children aged 3–15 years participated in the current study, of which 118 (29.2%) were MZ and 286 (70.8%) DZ twins. The mean age of participants was 9.63 in MZ twins and 9.47 in DZ twins. Table 1 shows the descriptive statistics of the participants.

**Table 1:** Descriptive statistics

*N* = 404			
Variable	Group	n	%
Zygosity	Monozygotic	118	29.2
	Dizygotic	286	70.8
Anterior Open Bite	No	381	94.3
	Yes	23	5.7
Mouth Breathing	No	315	78.0
	Yes	89	22.0
Atypical Swallowing	No	386	95.5
	Yes	18	4.5
Breastfeeding For 6 Months	No	133	34.6
	Yes	251	65.4
		M	SD
Bottle Feeding (Monthly)		21.37	± 15.569

One of the methods suggested for the discriminant validity of a PLS-SEM model is the HTMT (Heterotrait-Monotrait Ratio) criterion. If the HTMT values of the variables are below 0.90, it means that the discriminant validity is ensured [21]. The model provides discriminant validity as HTMT values ​​vary between 0.00 and 0.36. The model does not have multiple linear connection problems as all variable inflation factors (VIF) are found to be below 10 [22]. Since there is no linearity problem in the model, the path coefficients were examined in the second step (Table 2).

**Table 2:** Total and indirect effects

Total Effects				
	Original Sample (O)	Standard Deviation	T Statistics	*p*
Zygosity → Mouth Breathing	-0.202	0.054	3.743	0.000*
Mouth Breathing → Atypical Swallowing	0.218	0.065	3.365	0.001*
Atypical Swallowing → AOB	0.345	0.103	3.343	0.001*
Mouth Breathing → AOB	0.195	0.062	3.124	0.002*
Breastfeeding → AOB	-0.113	0.050	2.274	0.023*
Bottle Feeding → AOB	0.119	0.060	1.992	0.046*
Breastfeeding → Mouth Breathing	-0.086	0.051	1.696	0.090
Bottle Feeding → Atypical Swallowing	-0.064	0.040	1.586	0.113
Bottle Feeding → Mouth Breathing	0.025	0.048	0.525	0.600
Zygosity → AOB	-0.010	0.051	0.201	0.841
Breastfeeding → Atypical Swallowing	-0.009	0.050	0.183	0.855
Zygosity → Atypical Swallowing	0.001	0.053	0.017	0.986

Specific Indirect Effects				
Zygosity → Mouth Breathing → Atypical Swallowing	-0.044	0.018	2.488	0.013*
Mouth Breathing → Atypical Swallowing → AOB	0.075	0.032	2.336	0.020*
Zygosity → Mouth Breathing → Atypical Swallowing → AOB	-0.015	0.008	2.005	0.045*
Zygosity → Mouth Breathing → AOB	-0.024	0.014	1.700	0.089
Bottle Feeding → Atypical Swallowing → AOB	-0.024	0.016	1.475	0.140
Breastfeeding → Mouth Breathing → Atypical Swallowing	-0.019	0.013	1.460	0.144
Breastfeeding → Mouth Breathing → Atypical Swallowing → AOB	-0.006	0.005	1.305	0.192
Breastfeeding → Mouth Breathing → AOB	-0.010	0.009	1.201	0.230
Zygosity → Atypical Swallowing → AOB	0.016	0.021	0.756	0.450
Bottle Feeding → Mouth Breathing → Atypical Swallowing	0.005	0.011	0.500	0.617
Bottle Feeding → Mouth Breathing → Atypical Swallowing → AOB	0.002	0.004	0.484	0.629
Bottle Feeding → Mouth Breathing → AOB	0.003	0.007	0.445	0.657

*significant p value at 0.05 level. AOB: anterior open bite

### Pathways between the observed variables and AOB

#### The effect of zygosity on mouth breathing

The effect of zygosity on mouth breathing in the model was statistically significant (B=-0.202, t = 3.743, *p* = 0.000). For the zygosity variable, MZ twins were coded as 0 and DZ twins as 1. Therefore, the coefficient obtained shows the differentiation of DZ twinning compared to MZ twinning. The model predicted that the incidence of mouth breathing in DZ twins would be lower than in MZ twins. While the incidence of mouth breathing is 35.6% in 118 MZ twins, this rate decrease to 16.4% in 286 DZ twins cases. In addition, mouth breathing was seen in both siblings in 16 of 26 MZ twin pairs with mouth breathing (61.53%), while mouth breathing was detected in both siblings in 9 (23.68%) out of 38 DZ twin pairs with mouth breathing (Table 2).

#### The effect of mouth breathing on atypical swallowing

On the other hand, it was determined that mouth breathing effected that atypical swallowing (B = 0.218, t = 3.365, *p* = 0.001). While the incidence of atypical swallowing is 2.2% in 315 children without mouth breathing, this rate rises to 12.4% in 89 mouth breathing cases (Table 2).

#### The relation between zygosity, mouth breathing and atypical swallowing

Mouth breathing has a significant mediating effect between zygosity and atypical swallowing (B=-0.044, t = 2.488, *p* = 0.013). In other words, DZ twinning negatively affects mouth breathing and therefore indirectly lowers the level of atypical swallowing (Table 2).

#### The effect of atypical swallowing on AOB

Atypical swallowing triggered AOB (B = 0.345, t = 3.343, *p* = 0.001). While the incidence of AOB is 3.9% in 386 children without atypical swallowing, this rate rises to 44.4% in 18 atypical swallowing cases (Table 2).

#### The relation between mouth breathing, atypical swallowing, and AOB

Therefore, the atypical swallowing has a mediation effect between the other 2 disorders (B = 0.075, t = 2.336, *p* = 0.020). Mouth breathing causes atypical swallowing and therefore indirectly increases the likelihood of AOB. The total effect of mouth breathing on AOB was also statistically significant (B = 0.195, t = 3.124, *p* = 0.020). Approximately 38% of this positive effect occurs indirectly through atypical swallowing. Thus, atypical swallowing acts as a partial mediator variable between mouth breathing and AOB according to the PLS-SEM method (Table 2).

#### The effect of zygosity on AOB

The indirect effect of zygosity on AOB was statistically significant when the relations between these 4 variables were evaluated in a chain way (B=-0.015, t = 2.005, *p* = 0.045). Since the total effect of zygosity on AOB was insignificant (B=-0.010, t = 0.201, *p* = 0.841), it can be said that mouth breathing and atypical swallowing have full mediation effect between zygosity and AOB, jointly. However, when these two variables are evaluated separately, they do not play a mediating role alone between zygosity and AOB (*p* > 0.05). Nevertheless, it is worth emphasizing that the significance value of the mediating effect of mouth breathing is close to the 5% significant level (*p* = 0.085) (Table 2).

#### The effect of breastfeeding and bottle feeding on AOB

On the other hand, the total effects of breastfeeding and bottle feeding variables in the model on AOB are also significant. While breastfeeding decreases AOB incidence (B=-0.113, t = 2.274, *p* = 0.023), bottle feeding increases AOB incidence (B = 0.119, t = 0.1992 *p* = 0.046) (Table 2).

#### The effect of breastfeeding and bottle feeding on mouth breathing and atypical swallowing

According to the model, no significant effect of breastfeeding or bottle feeding on mouth breathing and atypical swallowing was detected (*p* > 0.05). Therefore, no indirect effects of these diets on atypical swallowing and AOB were found (*p* > 0.05). On the other hand, it is worth emphasizing that the p value of the protective effect of breastfeeding on mouth breathing is close to significance (*p* = 0.090) (Table 2).

#### The effect of zygosity on atypical swallowing and AOB

The effects of zygosity on atypical swallowing and AOB were also found to be statistically insignificant (*p* > 0.05) (Table 2). The adjusted R^2^ values of the model were calculated as 0.039 for atypical swallowing, 0.045 for mouth breathing, and 0.169 for AOB.

#### Logistic regression models

The research model was also analyzed separately with 3 logistic regression models. The first model is for mouth breathing probability, the second model is for atypical swallowing probability, and the third model is for estimating AOB probability. The results obtained are shared in Table 3.

**Table 3:** Results of logistic regression models

		Variables in the Equation					Omnibus Test	Nagelkerke R^2^
Regression Models	Dependent Variable	Predictor Variables	B	OR	S.E.	p	Model p	
**Model 1**	**Mouth Breathing**	**Constant**	− 0.408	0.665	0.325	0.209	0.000*	0.081
		**Zygosity**	**− 0.996**	**0.370**	**0.262**	0.000*		
		**Bottle Feeding**	0.004	1.004	0.008	0.631		
		**Breastfeeding**	− 0.497	0.608	0.264	0.059		
**Model 2**	**Atypical Swallowing**	**Constant**	**-3.696**	**0.025**	**0.936**	0.000*	0.000*	0.138
		**Zygosity**	0.238	1.269	0.600	0.691		
		**Bottle Feeding**	− 0.037	0.964	0.023	0.107		
		**Breastfeeding**	0.388	1.474	0.630	0.538		
		**Mouth Breathing**	**1.953**	**7.050**	**0.573**	0.001*		
**Model 3**	**AOB**	**Constant**	**-4.145**	**0.016**	**0.772**	0.000*	0.000*	0.265
		**Zygosity**	− 0.702	0.496	0.581	0.227		
		**Bottle Feeding**	**0.054**	**1.056**	**0.015**	0.000*		
		**Breastfeeding (Weekly)**	**-1.199**	**0.302**	**0.582**	0.040*		
		**Mouth Breathing**	0.716	2.047	0.589	0.224		
		**Atypical Swallowing**	**3.427**	**30.790**	**0.828**	0.000*		

B: Beta Cofficient, OR: Odds Ratio, S.E.: Standart Error, p: significant value, *significant p value at 0.05 level. AOB: anterior open bite

As a result of the logistic regression analysis, both models were found to be statistically significant (*p* = 0.00).

#### The first regression model

The effect of the zygosity variable on mouth breathing is statistically significant (B=-0.996, *p* = 0.000). For zygosity, MZ twins were coded as 0 and DZ twins as 1. Therefore, the coefficient obtained shows the differentiation of DZ twins compared to MZ twins. Accordingly, the probability of mouth breathing in DZ twins is 0.37 times lower than in MZ twins.

On the other hand, the effects of breastfeeding and bottle feeding variables on mouth breathing were statistically insignificant (*p* > 0.05). However, it should be considered that the significance value of breastfeeding is very close to the 5% significance level (*p* = 0.059).

The R^2^ value of the model was calculated as 0.081. Accordingly, the model can explain 8% of the variance in the probability of mouth breathing.

#### The second regression model

The effect of mouth breathing on atypical swallowing is statistically significant (B = 1.953, *p* = 0.001). The model estimates that mouth breathing in children increases the likelihood of atypical swallowing by 7.05 times.

Otherwise, the effects of zygosity, breastfeeding and bottle feeding variables on atypical swallowing were statistically insignificant (*p* > 0.05).

The R^2^ value of the model was calculated as 0.138. Accordingly, the model can explain approximately 14% of the variance in the probability of atypical swallowing.

#### The last and third regression model

The effect of atypical swallowing on AOB is statistically significant (B = 3.427, *p* = 0.000). The model estimates that atypical swallowing increases the likelihood of AOB by 30.79 times.

Also, the coefficients of breastfeeding and bottle feeding variables in the regression model for AOB are also significant. While breastfeeding reduces the probability of AOB (B=-1.199, *p* = 0.040), bottle feeding increases this probability (B = 0.054, t = 0.1992 *p* = 0.046). Breastfeeding for at least 6 months reduces the risk of AOB by 0.302 times, while bottle feeding for 1 month increases the risk of AOB 1.056 times.

Moreover, the effects of zygosity and mouth breathing variables on AOB were statistically insignificant (*p* > 0.05).

The R^2^ value of the third model was calculated as 0.265. Accordingly, the model can explain 26.5% of the variance in the AOB probability.

Using the coefficient and standard errors calculated in the models, the indirect effect of zygosity on atypical swallowing and the indirect effect of mouth breathing on AOB were examined with 3 different mediation tests (Table 4). Formulae for the tests provided here were drawn from MacKinnon and Dwyer [1993] [23] and from MacKinnon, Warsi, and Dwyer [1995] [24].

**Table 4:** Tests of Mediation effects

Zygosity → Mouth Breathing →Atypical Swallowing		
Tests	Test Statistic	*p*
**Sobel**	-2.538	0.011
**Aroian**	-2.490	0.012
**Goodman**	-2.588	0.009

Mouth Breathing → Atypical Swallowing → AOB		
Tests	Test Statistic	*p*
Sobel	2.631	0.009
Aroian	2.586	0.010
**Goodman**	2.678	0.007

*significant p value at 0.05 level. AOB: anterior open bite

#### The sobel tests

The z values ​​of Sobel (-2.538), Aroian (-2.490) and Goodman ( -2.588) were significant (*p* < 0.05); shows that the fully mediator variable feature of mouth breathing is statistically significant in the negative relation between zygosity and atypical swallowing. Also, the z values ​​of Sobel (3.025), Aroian (2.990) and Goodman (3.061) were significant (*p* < 0.05); shows that the partial mediator variable feature of atypical swallowing is statistically significant in the positive relationship between mouth breathing and AOB.

## Discussion

The main risk factors for AOB are harmful habits common among children, such as pacifier use, thumb sucking, bottle feeding, and atypical swallowing [5, 13]. In addition, genetic factors responsible for determining the facial skeletal pattern are also associated with the development and progression of this malocclusion type [25]. Although previous studies have evaluated the relationship between these risk factors and the occurrence of AOB, the causal pathways and direct and indirect relationships around these variables have not been explored in detail [26, 27].

In this study, prevalence of AOB was 5.7%, 6.8% in MZ twins and 5.2% in DZ twins. The prevalence of AOB was lower than the results of other studies on AOB [28, 29]. The relationship between AOB and pacifier sucking habit, which is one of the risk factors, has been examined and it has been reported that pacifier use has a direct effect on AOB [28]. This study examined the relationship between atypical swallowing, mouth-breathing, zygosity, breastfeeding and bottle feeding on AOB in terms of the total and indirect effects of these variables. The present study is the first to evaluate AOB in MZ and DZ twins. Thus, this study revealed the genetic similarity and the relationship between these factors.

The incidence of mouth breathing in MZ twin pairs was higher than in DZ twin pairs. It is seen in this study that genetic similarity increases the incidence of mouth breathing in twin pairs. This negative functional activity may affect the development of AOB by affecting facial features [30]. In the present study, it is seen that the effect of zygosity on mouth breathing has an indirect effect on AOB by increasing the frequency of atypical swallowing.

There is a relationship between the function of oro-facial muscles and facial structure, and between tongue position and AOB pattern [31]. In our study, a relationship was found between genetics and mouth breathing, mouth breathing and atypical swallowing, atypical swallowing and AOB. Similar results have been reported in other studies showing that tongue position during function has an effect on AOB [4, 32]. Some studies suggest that AOB is also associated with the habit of atypical swallowing. The inappropriate tongue posture during the swallowing movement influences and promotes the occurrence of this malocclusion [33, 34]. Aside from that, it is believed that an AOB is more likely to occur as a result of inadequate tongue position at rest than atypical swallowing patterns [10]. Individuals with an open bite, on the other hand, had almost similar pressure in all planes, lending support to the tongue’s adaptive functional hypothesis in an existing open bite [35]. It has also been reported that the relationship between AOB and atypical swallowing may be related to masseter hypotonia [36]. In this study it is seen that mouth breathing may have an effect on AOB by increasing atypical swallowing. Since the functions of orofacial muscles change during mouth breathing, it is thought that changes will occur in the facial structure and will also be effective on atypical swallowing. Another important point is that every patient with AOB due to mouth breathing will have tongue protrusion during swallowing. On this study contrary, some studies did not find a relationship between atypical swallowing and AOB [37].

Genetic and environmental influences promote vertical growth in the molar region, which is not compensated by growth in the condyle or posterior ramus [38]. In addition, proclination of anterior teeth with bottle feeding and non-nutritive sucking habits such as finger and pacifier sucking can also have an effect on AOB with normal molar height [39]. In this study, it is seen that feeding with a bottle has an increasing effect on the frequency of AOB. Brestfeeding has a reducing effect on the frequency of AOB. Among the nutritional forms, breastfeeding ensures the proper development of the stomatognathic system by working the oro-facial muscles. These positive functional activities have an impact on the development of healthier habits in terms of oral health [13].

The study had some limitations that should be considered. Limitations of this study may include that the diagnosis of mouth breathing was not made by an otorhinolaryngologist and multidisciplinary team. In addition, the inability to differentiate the diagnosis of AOB as skeletal or dental due to the inability to obtain cephalometric radiography from the patients can be considered as another limiting factor. Another limitation was the the possibility that the answers given by the parents, such as sucking times, could be incomplete.

This study has important aspects to consider. First, this study is a twin study in which genetic similarity was also examined. In addition, this study strengthened the causality between these factors by evaluating different risk factors related to diet, bad oral habits and AOB. Therefore, it is important to investigate different determinants that may have an effect on malocclusion formation. In addition, studies in children are extremely important because bad oral habits that lead to the development of malocclusion can be maintained throughout life. This study identified the relationships between different factors and the presence of AOB, thus contributing to the establishment of a path to determine the roles these factors play in the etiology of AOB.

In conclusion, the findings of this study demonstrate in detail the relationships between AOB and zygosity, atypical swallowing, mouth breathing, breastfeeding and bottle feeding. The prevalence of mouth breathing in MZ twin sibling pairs is higher than in DZ twin pairs. The effect of zygosity on mouth breathing appears to have an indirect effect on AOB by increasing the frequency of atypical swallowing. In our study, a direct relationship was found between genetics and mouth breathing, mouth breathing and atypical swallowing, atypical swallowing and AOB. Additionally, it appears that mouth breathing may have an effect on the AOB by increasing atypical swallowing. Since the functions of the oro-facial muscles change in mouth breathing, it is thought that changes in the facial structure will occur and will also affect atypical swallowing. It has also been determined that bottle feeding has an increasing effect on the frequency of AOB. Among the nutritional methods, brestfeeding has been found to have a reducing effect on the frequency of AOB by ensuring the proper development of the orofacial muscles and the stomatognathic system.

## Data Availability

No datasets were generated or analysed during the current study.

## References

[1] Silvestrini-Biavati A, Salamone S, Silvestrini-Biavati F, Agostino P, Ugolini A (2016) Anterior open-bite and sucking habits in Italian preschool children. Eur J Pediatr Dent 17(1):43–46 26949238

[2] Rijpstra C, Lisson JA (2015) Etiology of anterior open bite-a review. J Orofac Orthop 77:281–286 10.1007/s00056-016-0029-1 27098640

[3] Ruiz Gutierrez DA, Garzon JS, Franco JQ, Botero-Mariaca P (2021) Anterior open bite and its relationship with dental arch dimensions and tongue position during swallowing and phonation in individuals aged 8–16 years: a retrospective case-control study. Int Orthod 19:107–116 33518486 10.1016/j.ortho.2020.12.005

[4] Dimberg L, Lennartsson B, Soderfeldt B, Bondemark L (2013) Malocclusioms in children at 3 and 7 years of age: a longitudinal study. Eur J Orthod 35(1):131–137 22045694 10.1093/ejo/cjr110

[5] Izi-Iyamu IN, Isiekwe MC (2012) Prevalence and factors associated with anterior open bite in 2 to 5 year old children in Benin city. Nigeria Afr Health Sci 12(4):446–451 23513076 10.4314/ahs.v12i4.8 PMC3598284

[6] Lin LH, Huang GW, Chen CS (2013) Etiology and treatment modalities of anterior open bite malocclusion. J Exp Clin Med 5(1):1–4

[7] Peres KG, Latorre MR, Sheiham A, Peres M, Victora C, Barros F (2006) Social and biological early life infuences on the prevalence of open bite in Brazilian 6-years old. Int J Paediatr Dent 17(1):41–49 10.1111/j.1365-263X.2006.00793.x 17181578

[8] Hsu JY, Cheng JHC, Feng SW, Lai PC, Yoshida N, Chiang PC (2024) Strategic treatment planning for anterior open bite: a comprehensive approach. J Dent Sci 19(3):1328–1337 39035309 10.1016/j.jds.2024.04.001 PMC11259669

[9] Lone IM, Zohud O, Midlej K, Paddenberg E, Krohn S, Kirschneck C et al (2023) Anterior Open Bite Malocclusion: from clinical treatment strategies towards the dissection of the genetic bases of the Disease using human and Collaborative Cross Mice Cohorts. J Personalized Med 13(11):1617 10.3390/jpm13111617 PMC10672619 38003932

[10] Artese F, Fernandes LQP, de Oliveira Caetano SR, Miguel JAM (2023) Early treatment for anterior open bite: choosing adequate treatment approaches. Semin Orthod 29:207–215

[11] Vasconcelos FM, Massoni AC, Heimer MV, Ferreira A, Katz RT, Rosenblatt A (2011) Non-nutritive sucking habits, anterior open bite and associated factors in Brazilian children aged 30–59 months. Braz Dent J 22(2):140–145 21537588 10.1590/s0103-64402011000200009

[12] Charchut SW, Allred EN, Needleman HL (2003) The effects of infant feeding patternes on the occlusion of the primary dentition. J Dent Child 70(3):197–203 14998201

[13] Katz CRT, Rosenblatt A (2005) Non nutritive sucking habits and anterior open bite in Brazilian children: a longitudinal study. Pediatr Dent 27(5):369–373 16435635

[14] Heimer MV, Katz CRT, Rosenblatt A (2008) Non-nutritive sucking habits, dental malocclusions, and facial morphology in Brazilian children: a longitudinal study. Eur J Orthod 30(6):580–585 18775881 10.1093/ejo/cjn035

[15] Knösel M, Nüser C, Jung K, Helms HJ, Engelke W, Sandoval P (2016) Interaction between deglutition, tongue posture, and malocclusion: a comparison of intraoral compartment formation in subjects with neutral occlusion or different types of malocclusion. Angle Orthod 86(5):697–705 26894981 10.2319/101615-699.1 PMC8600850

[16] Machado DB, Brizon VS, Ambrosano GM, Madureira DF, Gomes VE, de Oliveira AC (2014) Factors associated with the prevalence of anterior open bite among preschool children: a population-based study in Brazil. Dent Press J Orthod 19(5):103–109 10.1590/2176-9451.19.5.103-109.oar PMC4296660 25715723

[17] Hair JF, Ringle CM, Sarstedt M (2011) PLS-SEM: indeed a silver bullet. J Market Theory Pract 19(2):139–152

[18] Gómez-González C, González-Mosquera A, Alkhraisat MH, Anitua E (2024) Mouth Breathing and its impact on atypical swallowing: a systematic review and Meta-analysis. Dentistry J 12(2):21 10.3390/dj12020021 PMC10888353 38392225

[19] Hair J Jr, Sarstedt M, Hopkins L, Kuppelwieser VG (2014) Partial Least Squares Structural Equation Modeling (PLS-SEM): an Emerging Tool in Business Research. Eur Busin Rev 26(2):106–112

[20] Preacher KJ, Leonardelli GJ (2010) Calculation fort he sobel test. [accessed 2022 Dec 14]. http://www.quantpsy.org/sobel/sobel.htm

[21] Henseler J, Ringle C, Sarsted M (2015) A new criterion for assesing discriminant validity in variance-based structural equation modeling. J Acad Market Sci 43:115–135

[22] Hair J, Black W, Babin B, Anderson R, Tatham R (2006) Multivariate data analysis. Pearson Prentice Hall, Upper Saddle River, NJ

[23] MacKinnon DP, Dwyer JH (1993) Estimating mediated effects in prevention studies. Evaluat Rev 17(2):144–158

[24] MacKinnon DP, Warsi G, Dwyer JH (1995) A simulation study of mediated effect measures. Multiv Behav Res 30(1):41–62 10.1207/s15327906mbr3001_3 PMC2821114 20157641

[25] González MX, Mafla AC (2016) Occlusal and cephalometric characteristics of anterior open-bite among Colombian 5–10 years old school children. J Oral Res 5(6):232–239

[26] De Sousa RV, Lima G, Ribeiro A, Targino R (2014) Prevalence and associated factors for the development of anterior open bite and posterior crossbite in the primary dentition. Braz Dent J 25(4):336–342 25250499 10.1590/0103-6440201300003

[27] Lima AADSJ, Alves CMC, Ribeiro CCC, Pereira ALP, da Silva AAM, Silva LFGE, Thomaz EBAF (2017) Effects of conventional and orthodontic pacifiers on the dental occlusion of children aged 24–36 months old. Int J Paediatr Dent 27(2):108–119 26856705 10.1111/ipd.12227

[28] Moraes RB, Knorst JK, Pfeifer ABR, Vargas-Ferreira F, Ardenghi TM (2021) Pathways to anterior open bite after changing of pacifier sucking habit in preschool children: a cohort study. Int J Paediatr Dent 31(2):278–284 32949057 10.1111/ipd.12725

[29] Gomes MC, Neves ETB, Perazzo MF, Martins CC, Paiva SM, Granville-Garcia AF (2018) Association between psychological factors, socio-demographic conditions, oral habits and anterior open bite in five-year-old children. Acta Odont Scand 76(8):553–558 29764280 10.1080/00016357.2018.1472294

[30] Laudadio C, Inchingolo AD, Malcangi G, Limongelli L, Marinelli G, Coloccia G, Montenegro V, Patano A, Inchingolo F, Bordea IR et al (2021) Management of anterior open-bite in the deciduous, mixed and permanent dentition stage: a descriptive review. J Biol Regul Homeost Agents 35(2):271–281 34281324 10.23812/21-2supp1-27

[31] Pisani L, Bonaccorso L, Fastuca R, Spena R, Lombardo L, Caprioglio A (2016) Systematic review for orthodontic and orthopedic treatments for anterior open bite in the mixed dentition. Prog Orthod 17(1):28 27615261 10.1186/s40510-016-0142-0 PMC5027197

[32] Fujiki T, Inoue M, Miyawaki S, Toshikazu Nagasaki KT, Takano-Yamamoto T (2004) Relationship between maxilofacial morphology and deglutitive tongue movement in patients with anterior open bite. Am J Orthod Dentofac Orthop 125(2):160–167 10.1016/j.ajodo.2003.03.009 14765053

[33] Maciel CT, Leite IC (2005) Aspectos etiológicos Da mordida aberta anterior e suas implicações Nas funções orofaciais. Pro Fono 17(3):293–302 16389786 10.1590/s0104-56872005000300003

[34] Marcomini L, Santamaria M Jr, Lucato AS, Santos JCB, Tubel CAM (2010) Prevalence of malocclusion and its relationship with functional changes in the breathing and in the swallowing. Braz Dent Sci 13(1/2):52–58

[35] Kurihara K, Fukui T, Sakaue K, Hori K, Ono T, Saito I (2019) The effect of tongue thrusting on tongue pressure production during swallowing in adult anterior open bite cases. J Rehabil 46(10):895–902 10.1111/joor.12820 31081951

[36] Maspero C, Prevedello C, Giannini L, Galbiati G, Farronato G (2014) Atypical swallowing: a review. Minerva Stomatol 63(6):217–227 25267151

[37] de Lemos CM, de Souza Junqueira PA, Gomez MVSG, de Faria MEJ, de Cássia Basso S (2006) Study of the relationship between the dentition and the swallowing of mouth breathers. Int Arch Otorhinplaryngol 10:370

[38] Daer AA, Abuaffan AH (2016) Skeletal and dentoalveolar cephalometric features of anterior open bite among Yemeni adults. Scientifica 2016:1–5 10.1155/2016/3147972 PMC4844903 27190680

[39] Schmid KM, Kugler R, Nalabothu P, Bosch C, Verna C (2018) The effect of pacifier sucking on orofacial structures: a systematic literature review. Prog Orthod 19(8):1–11 29532184 10.1186/s40510-018-0206-4 PMC5847634

